# Paraquat and MPTP induce neurodegeneration and alteration in the expression profile of microRNAs: the role of transcription factor Nrf2

**DOI:** 10.1038/s41531-017-0033-1

**Published:** 2017-10-20

**Authors:** Qingqing Wang, Nan Ren, Zhipeng Cai, Qingxia Lin, Zhangjing Wang, Qunwei Zhang, Siying Wu, Huangyuan Li

**Affiliations:** 1 Department of Preventive Medicine, Fujian Provincial Key Laboratory of Environment Factors and Cancer, School of Public Health, Fujian Medical University, 350122 Fuzhou, China; 2 Zhangzhou Entry-Exit Inspection and Quarantine Bureau, Shicangduan, Shuixian Street, 363000 Zhangzhou, China; 3 Department of Environmental and Occupational Health Sciences, University of Louisville, 485 E. Gray Street, Louisville, KY 40202 USA; 4 Department of Epidemiology and Health Statistics, Fujian Provincial Key Laboratory of Environment Factors and Cancer, School of Public Health, Fujian Medical University, 350122 Fuzhou, China

## Abstract

Both transcription factors (TFs) and microRNAs (miRNAs) can exert a widespread impact on gene expression. In the present study, we investigated the role of Nrf2 in paraquat-induced intracorporeal neurodegeneration and miRNA expression by exposing Nrf2 wild-type and knockout mice to paraquat (PQ) or 1-methyl-4-phenyl-1,2,3,6-tetrahydropyridine (MPTP). Exposure to 10 mg/kg PQ or 30 mg/kg MPTP caused damage to nerve cells in the substantia nigra (SN) in both Nrf2 (+/+) and Nrf2 (−/−) ICR mice, which included cell morphological changes, detectable apoptosis and a significant reduction in the number of dopaminergic (DA) neurons. When mice were exposed to the same PQ dose of 10 mg/kg, significant fewer tyrosine hydroxylase (TH)-positive DA neurons were observed in the Nrf2 (−/−) mice than that in the Nrf2 (+/+) mice. Both Nrf2 deficiency and PQ or MPTP exposure could alter miRNA expression profile in the SN, suggesting the potential involvement of Nrf2 in the PQ-induced or MPTP-induced miRNA expression alteration. The expression of miR-380-3p was altered by the Nrf2-MPTP interaction effect. miR-380-3p/Sp3-mRNA pathway is likely part of the mechanism of MPTP-induced neurodegeneration. Collectively, our results corroborated the protective role of Nrf2 and also demonstrated the essential interaction of Nrf2 with miRNAs in intracorporal neurodegeneration induced by neurotoxicants.

## Neurodegeneration: Uncovering protective mechanisms

The gene expression regulators Nrf2 and small regulatory RNA molecule miR-380-3p protect nerve cells from damage caused by neurotoxins. Huangyuan Li, at Fujian Medical University, China, and colleagues examined the effects of paraquat and *MPTP* (1-methyl-4-phenyl-1, 2, 3, 6-tetrahydropyridine), two chemicals known to induce parkinsonism, in mice lacking Nrf2, a key mediator of antioxidant and anti-inflammatory responses. They found that after MPTP or paraquat exposure, the extent of neuronal loss in the substantia nigra was greater in mice lacking Nrf2 than in wild-type controls. Moreover, Nrf2 deficiency prevented an upregulation of miR-380-3p following MPTP exposure. These findings implicate miR-380-3p in the mechanism through which Nrf2 protects the brain from Parkinson’s disease-related cell death and opens new avenues of investigation for developing more effective neuroprotective therapies.

## Introduction

Parkinson’s disease (PD) is characterized by a progressive and selective loss of dopaminergic neurons in the substantia nigra (SN), which plays an important role in normal motor function. Paraquat (PQ) is a nonselective herbicide widely used in worldwide agricultural practices. The chemical structure of PQ is similar to the active metabolite of 1-methyl-4-phenyl-1,2,3,6-tetrahydropyridine (MPTP) in the body (MPP^+^, 1-methyl 4-phenylpyridine). Both PQ and MPTP are two types of common neurotoxicants, which can cause neurodegeneration.

Nuclear factor erythroid 2-related factor 2 (Nrf2) is a redox-sensitive master regulatory transcription factor, which can bind to the antioxidant response element in the promotor region of antioxidant enzymes such as heme oxygenase 1 (HO-1), glutamate cysteine ligase catalytic subunit, and NAD(P)H dehydrogenase quinone 1, thus regulating their expression.^1–3^ These enzymes are involved in antioxidant response and detoxification reactions. Nrf2 has been found in most brain cells including microglia, astrocytes and dopaminergic neurons. A number of recent studies suggest that as well as regulating MPTP toxicity, Nrf2 may also regulate PQ toxicity.^4–6^ Our previous research showed that PQ caused nerve cell damage and induced apoptosis in PC12 cells, and also caused upregulation of miR-133b.^7^ Pre-treatment of dopaminergic cells with tert-butyl hydroquinone (*t*BHQ) protected cells from PQ-induced neurodegeneration.^8^ These studies indicate that Nrf2 plays an important role in the development of neurodegeneration. Therefore, the effects of PQ and MPTP on Nrf2 (+/+) and Nrf2 (−/−) ICR mice were studied here, including to study the morphological changes of the cells from the SN, and apoptosis and Nrf2 protein level in tyrosine hydroxylase (TH)-labeled dopamine (DA) neurons. The potential role of Nrf2 in PQ/MPTP-induced damage in nerve cells was also studied.

It is known that miRNAs and transcription factors are the two main types of trans-acting factors. They have a close relationship in the regulation network, and make up the feedback loop that regulates gene expression.^9^ They also play an important role in the progression of neurodegenerative diseases and a variety of cellular processes (e.g., cell differentiation).^9–12^ For example, miR-133b negatively regulates a crucial transcription factor, Pitx3, in midbrain dopaminergic neurons.^12^ The close association between miRNAs and Nrf2 transcription factor has also been found in breast cancer^13,14^ and pure sickle cell disease.^15^ A study in SH-SY5Y cells (a human derived cell line) was the first to identify and verify that the Nrf2 gene is the target gene of miR-153/miR-27a/miR-142-5p/miR-144.^16^ However, detailed association between miRNA and Nrf2 is yet to be determined. Several questions have yet to be answered, including whether there are links between the expression of miRNAs in nerve cells and Nrf2, and whether Nrf2 influences the role of miRNAs in PQ-induced or MPTP-induced neurodegeneration.

In a word, the present study sought to further confirm the role of Nrf2 in PQ-induced or MPTP-induced neurodegeneration, to perform a preliminary analysis on the role of Nrf2 on miRNAs involved in PQ-induced or MPTP-induced nerve cell damage, and to explore specific miRNA-targeted genes involved in PQ-induced or MPTP-induced neurodegeneration. Using Nrf2 knockout mouse (loss-of-function phenotype) model, we investigated neurodegeneration and the changes in miRNA expression after exposure to PQ or MPTP and how this is influenced by Nrf2. Our results may provide evidence on underlying mechanisms of PQ-induced or MPTP-induced neurodegeneration, and berrantly expressed miRNA may be useful in further investigation to detect early symptoms of PQ-induced or MPTP-induced neurodegeneration. Understanding the role of transcription factor Nrf2 on neurodegeneration and alteration in the expression profile of microRNAs induced by PQ or MPTP in vivo may provide insights into more effective preventive and therapeutic approaches for the PD.

## Results

### The establishment of PD model by exposure of Nrf2 (+/+) mice to PQ or MPTP

#### Hematoxylin and eosin (HE) staining

**Fig. 1:**
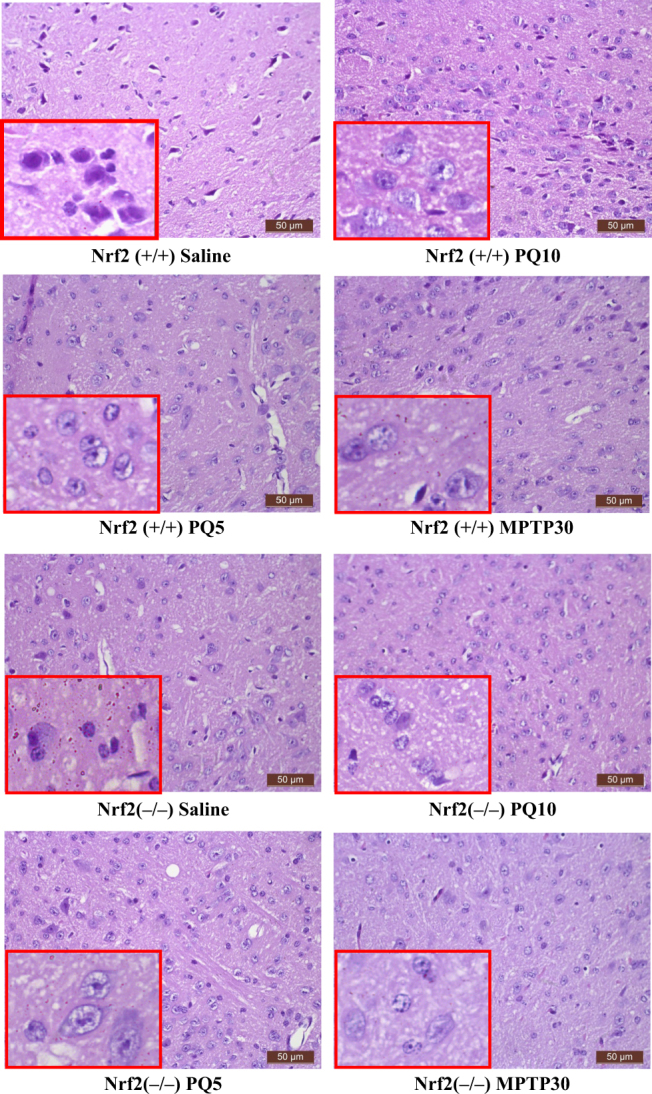
HE staining in the SN of Nrf2 (+/+) and Nrf2 (−/−) mice after treatment with PQ, MPTP and saline for neuropathological changes. Each microgram (100×) contains a view of SN from each group (*n* = 3). Representative damaged cells were shown in bottom left corner of the panel (400×)

As compared to the control group, Nrf2 (+/+) ICR mice exposed to MPTP at 30 mg/kg exhibited neural cell damage, which was reflected by dark staining (blue–black) of cell nuclei, karyopyknosis and nucleus fragmentation (Fig. 1). Similarly, neural cells in 5 mg/kg PQ group or 10 mg/kg PQ group also showed karyopyknosis, fragmentation and a blue–black nucleus.

#### TH immunohistochemical staining

**Fig. 2:**
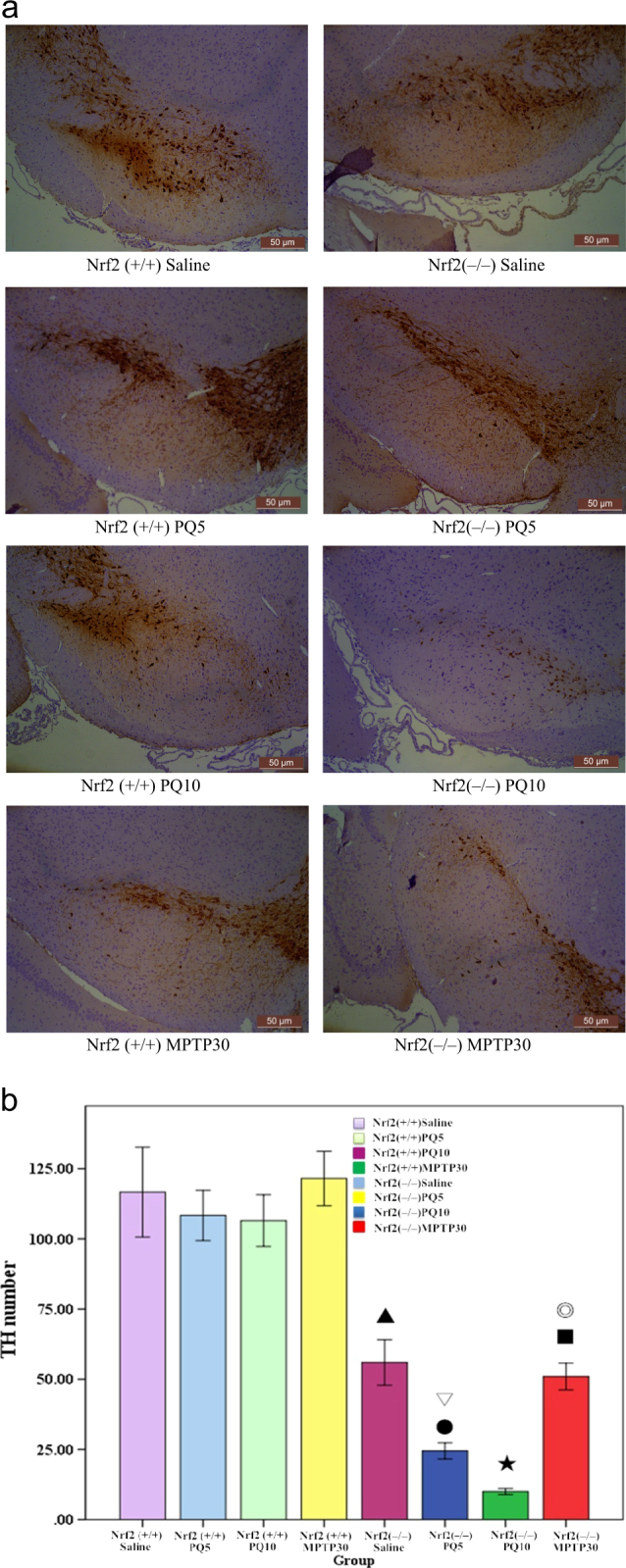
Neuropathological changes in the SN of Nrf2 (+/+) or Nrf2 (−/−) mice after treatment with PQ, MPTP or saline (*n* = 3). **a** Each microgram (100×) shows a representative perspective view of SN immunostained with TH from each group. **b** The loss of TH-positive DA neurons induced by PQ or MPTP in each group, reflected by numbers of residual neurons. ▲ Nrf2 (+/+) PQ10 vs. Nrf2 (+/+) saline (*p* < 0.01), ★ Nrf2 (+/+) MPTP30 vs. Nrf2 (+/+) saline (*p* < 0.01), ● Nrf2 (−/−) PQ10 vs. Nrf2 (−/−) saline (*p* < 0.01), ■ Nrf2 (−/−) MPTP30 vs. Nrf2 (−/−) saline (*p* < 0.01), ▽ Nrf2 (−/−) PQ10 vs. Nrf2 (+/+) PQ10 (*p* < 0.05), ◎ Nrf2 (−/−) MPTP30 vs. Nrf2 (+/+) MPTP30 (*p* < 0.05)

Using immunohistochemical staining technique to stain TH protein positive DA neurons, we explored the PQ-induced or MPTP-induced alteration in DA neurons (TH). As shown on the left of Fig. 2a, Nrf2 (+/+) ICR mice exposed to MPTP had a significantly lower number of DA neurons compared to the control group (*p* < 0.05). In mice exposed to PQ at 10 mg/kg, the number of DA neurons was significantly smaller than that in the control group (*p* < 0.01, Fig. 2b). However, the number of DA neurons in mice exposed to PQ at 5 mg/kg was not significantly different from that in the control group (*p* > 0.05, Fig. 2b).

#### Cell apoptosis detected by in situ nick end labeling (TUNEL)

**Fig. 3:**
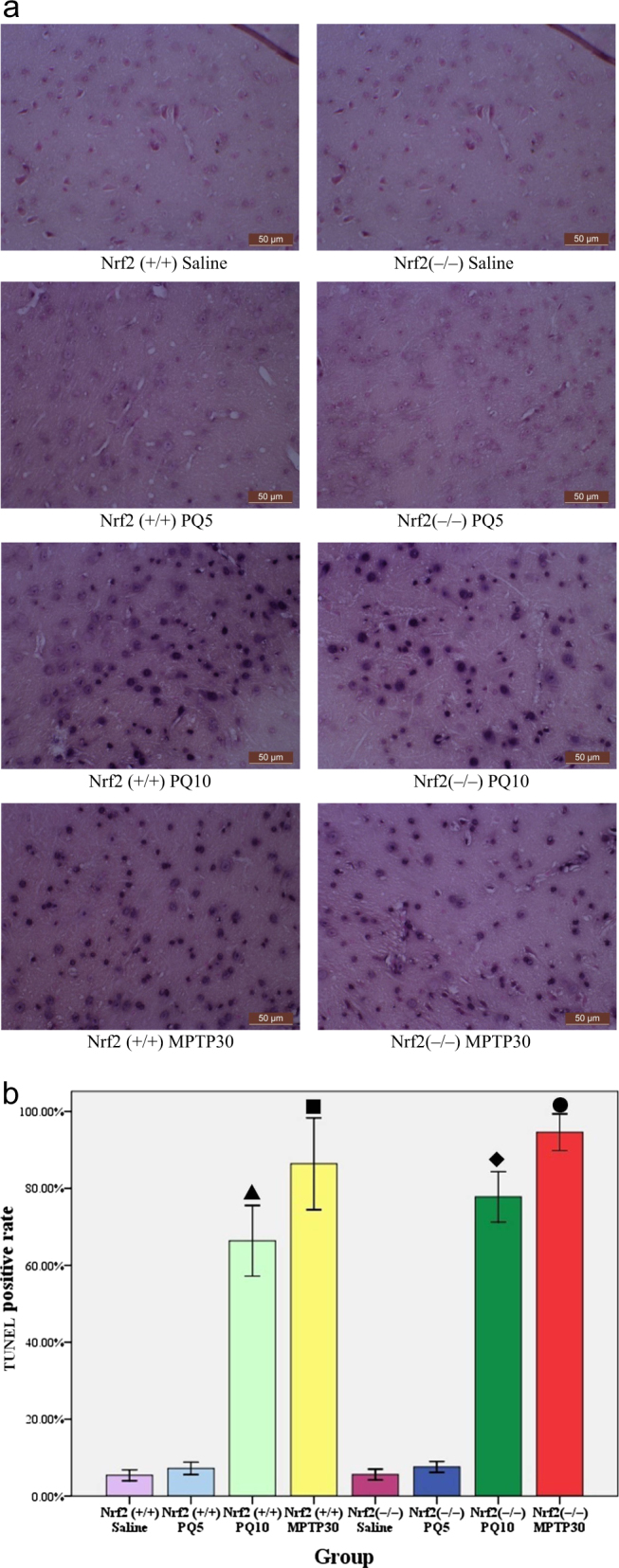
Cell apoptosis in the SN of Nrf2 (+/+) and Nrf2 (−/−) mice after treatment with PQ, MPTP or saline (*n* = 3). **a** Each microgram (400×) shows a representative view of apoptosis in the SN of each group. **b** TUNEL-positive rate in each group. We counted the number of TUNEL staining positive cells and the number of total cells, and calculated the TUNEL staining positive rate (the number of TUNEL positive cells/total number of cells x 100%). ▲ Nrf2 (+/+) PQ10 vs. Nrf2 (+/+) saline (*p* < 0.01), ■Nrf2 (+/+) MPTP30 vs. Nrf2 (+/+) saline (*p* < 0.01), ◆Nrf2 (−/−) PQ10 vs. Nrf2 (−/−) saline (*p* < 0.01), ●Nrf2 (−/−) MPTP30 vs. Nrf2 (−/−) saline (*p* < 0.01)

As shown on the left of Fig. 3, the SN in Nrf2 (+/+) ICR mice showed cell apoptosis after MPTP treatment at 30 mg/kg and also after PQ treatment at 10 mg/kg. However, there was no obvious apoptosis after PQ treatment at 5 mg/kg.

### Neuropathological changes in SN tissue of Nrf2 (−/−) ICR mice after exposure to PQ or MPTP

#### HE staining

In Nrf2 (−/−) ICR mice exposed to MPTP at 30 mg/kg or PQ at either 5 or 10 mg/kg, the nuclei of neural cells appeared blue–black and showed karyopyknosis and fragmentation compared to that in the control group (shown on the lower left panel in Fig. 1).

#### Immunohistochemical staining of TH protein in DA neurons

As shown on the right of Fig. 2a, there were significantly fewer DA neurons in Nrf2 (−/−) ICR mice exposed to MPTP than in the control group (*p* < 0.01). In terms of PQ exposure, there was no significant difference between the 5 mg/kg group and the control group (*p* > 0.05). However, when the dose rose to 10 mg/kg PQ, there was a significant reduction in the number of DA neurons (*p* < 0.01), as shown in Fig. 2b.

#### Detection of apoptosis with in situ nick end-labeling (TUNEL)

As shown on the right of Fig. 3, the SN in Nrf2 (−/−) ICR mice showed cell apoptosis after MPTP treatment at 30 mg/kg compared to the control group (*p* < 0.01). After PQ treatment at 5 mg/kg, there was no visible apoptosis, but on increasing the dose to 10 mg/kg, there was apparent apoptosis in the SN (*p* < 0.01), as shown in Fig. 3b.

### The morphological and quantitative changes of DA neurons and cell apoptosis in the SN tissue of Nrf2 (+/+) or Nrf2 (−/−) ICR mice after exposure to PQ or MPTP

In the SN of Nrf2 (+/+) or Nrf2 (−/−) ICR mice treated with saline, there was no significant difference from the control group in terms of cell morphology, apoptosis and the number of TH-positive DA neurons (*p* > 0.05), as shown in Fig. 2a, b.

In Nrf2 (+/+) or Nrf2 (−/−) ICR mice exposed to PQ at 5 mg/kg, no significant cell apoptosis was found in the SN, but the nuclei showed condensation, fragmentation and were blue–black in color (Fig. 1). There was no significant difference in the number of DA neurons in either Nrf2 (+/+) or Nrf2 (−/−) ICR mice, measured by immunohistochemical staining of the TH protein, compared to the control group (*p* > 0.05, Fig. 2a, b). In Nrf2 (+/+) and Nrf2 (−/−) ICR mice exposed to MPTP at 30 mg/kg, there was evidence of cell apoptosis (Fig. 3) and the nuclei showed condensation, fragmentation and appeared blue–black (Fig. 1). Similarly, in the SN of Nrf2 (+/+) or Nrf2 (−/−) ICR mice exposed to PQ at 10 mg/kg, there was obvious cell apoptosis (Fig. 3), along with blue–black nuclei in a condensed and fragmented form (Fig. 1). Although both were exposed to the same PQ dose of 10 mg/kg, there were significantly fewer TH-positive DA neurons in Nrf2 (−/−) ICR mice than in Nrf2 (+/+) group (*p* < 0.05, Fig. 2a, b).

### Expression level of the Nrf2 protein in SN tissue of Nrf2 (+/+) ICR or Nrf2 (−/−) ICR mice after treatment with PQ or MPTP

#### Comparison of the expression level of Nrf2 protein between Nrf2 (−/−) and Nrf2 (+/+) mice

**Fig. 4:**
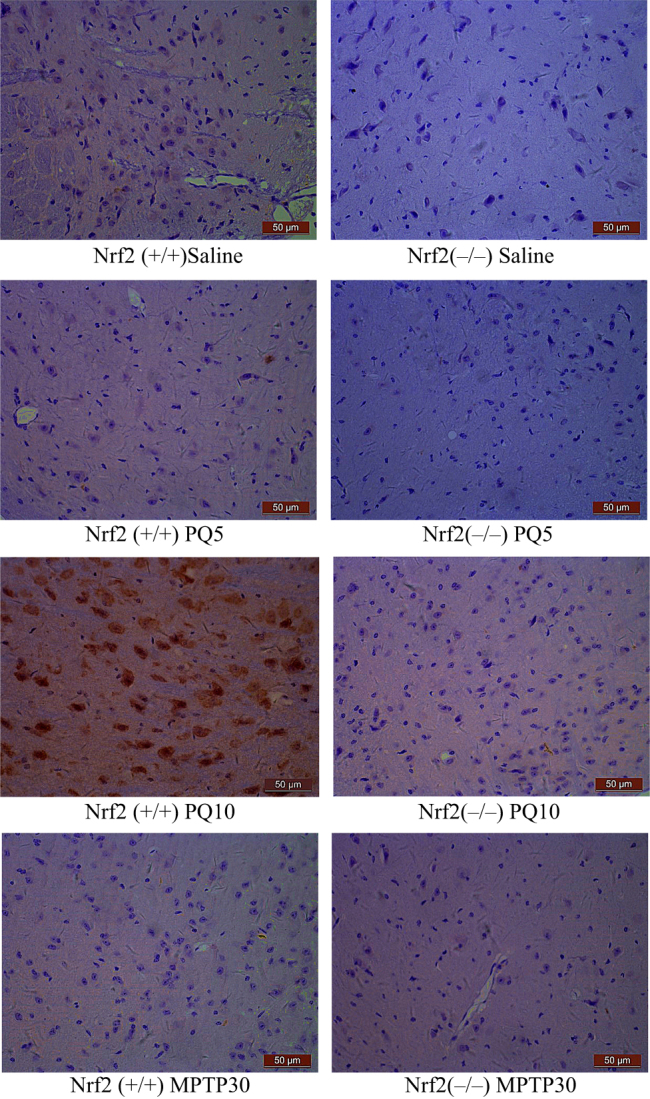
Expression of Nrf2 protein in the SN of Nrf2 (+/+) or Nrf2 (−/−) mice after treatment with PQ, MPTP or saline (*n* = 3). Each microgram shows a representative view of an Nrf2-immunostained slice from the SN (400×). Nrf2 protein staining has confirmed results of both Nrf2 knockout and response of Nrf2 to PQ/MPTP exposure in mice

As shown in Fig. 4, when mice were exposed to saline, the expression level of Nrf2 protein in SN tissue of Nrf2 (−/−) ICR mice was significantly (*p* < 0.05) lower than that in Nrf2 (+/+) ICR mice.

#### Expression level of Nrf2 protein in Nrf2 (+/+) ICR mice after treatment with MPTP and PQ

Expression of Nrf2 protein was lower in mice exposed to MPTP at 30 mg/kg or PQ at 5 mg/kg compared to mice treated with saline. However, expression of Nrf2 increased in mice exposed to PQ at 10 mg/kg (*p* < 0.05, Fig. 4).

### Alteration in the expression profile of microRNAs in SN tissue of Nrf2 (+/+) ICR and Nrf2 (−/−) ICR mice after treatment with PQ or MPTP

First, we analyzed the link between transcription factor Nrf2 and miRNA expression. We then analyzed changes in miRNA expression profile after exposure to PQ or MPTP in WT mice. Finally, we analyzed the relationship between transcription factor Nrf2 and miRNA expression profile in Nrf2 knockout mice after exposure to PQ or MPTP. The overall aim was to investigate the effects of PQ or MPTP on the regulatory network of Nrf2 and miRNA. Alteration in the expression profile of microRNAs in SN tissue of Nrf2 (+/+) ICR or Nrf2 (−/−) ICR mice after treatment with PQ or MPTP was shown in Table 3.

### The expression intensity of miR-380-3p in the SN of Nrf2 (+/+) ICR mice after PQ or MPTP treatment

**Fig. 5:**
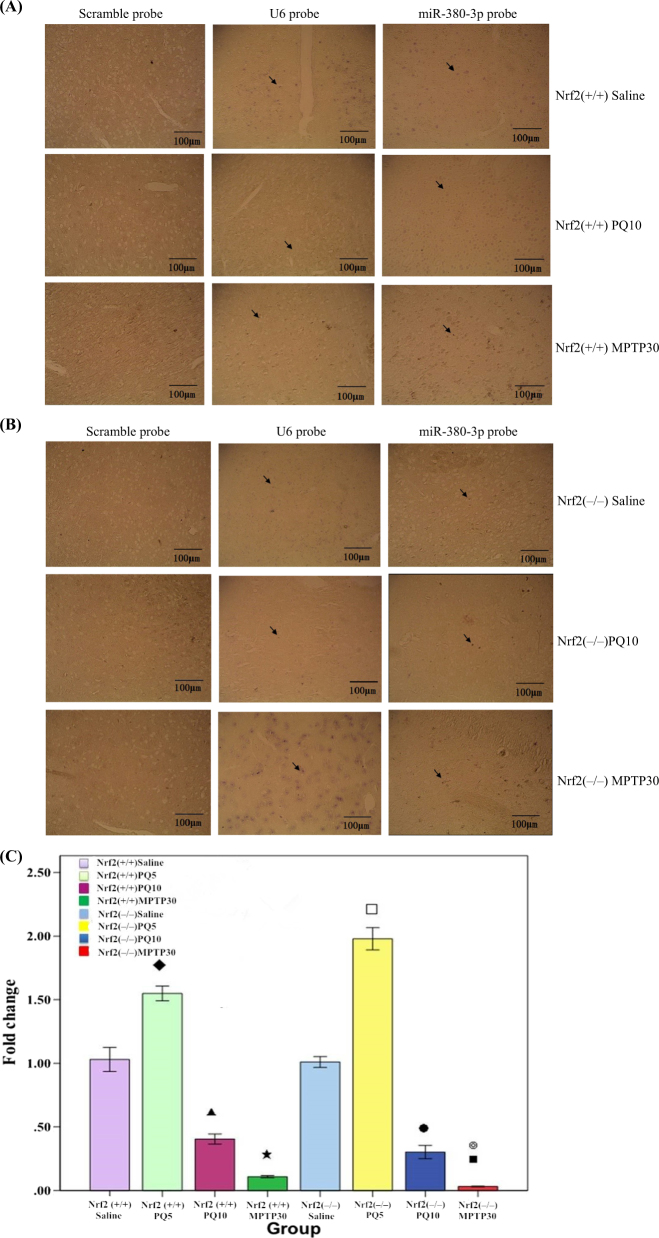
The expression level of miR-380-3p in the SN of Nrf2 (+/+) and Nrf2 (−/−)after treatment with PQ, MPTP or saline. The arrows indicated the signal of LNA™ probes for miR-380-3p. **a** The expression intensity of miR-380-3p analyzed by LNA–ISH hybridization in the SN of Nrf2 (+/+) ICR mice after saline, PQ or MPTP treatment (*n* = 3); each microgram (400×) shows a representative view of a slice of the SN from each group. **b** The expression intensity of miR-380-3p analyzed by LNA–ISH hybridization in Nrf2 (−/−) ICR mice SN after saline, PQ or MPTP treatment (*n* = 3); each microgram (400×) shows a representative view of a slice of the SN from each group. **c** The expression levels of miR-380-3p analyzed by real-time PCR in Nrf2 (+/+) and Nrf2 (−/−) SN after treatment with PQ, MPTP or saline (*n* = 4). ◆ Nrf2 (+/+) PQ5 vs. Ap (*p* < 0.01), ▲ Nrf2 (+/+) PQ10 vs. Nrf2 (+/+) saline (*p* < 0.01), ★ Nrf2 (+/+) M*P*TP30 vs. Nrf2 (+/+) saline (*p* < 0.01), □Nrf2 (−/−) PQ5 vs. Nrf2 (−/−) saline (*p* < 0.01), ●Nrf2 (−/−) PQ10 vs. Nrf2 (−/−) saline (*p* < 0.01), ■ Nrf2 (−/−) MPTP30 vs. Nrf2 (−/−) saline (*p* < 0.01), ◎ Nrf2 (−/−) MPTP30 vs. Nrf2 (+/+) MPTP30 (*p* < 0.01)

As shown in Fig. 5a, both PQ treatment at 10mg/kg and MPTP treatment at 30 mg/kg reduced the expression intensity of miR-380-3p in the SN of Nrf2 (+/+) ICR mice. The result is consistent with miRNA expression profile (Table 1).

**Table 1:** The miR-380-3p expression level in the substantia nigra of Nrf2 (+/+) and Nrf2 (−/−) mice after treatment with PQ, MPTP or saline (≥1.5 fold, *p* < 0.05)

Contrast	Fold Change	*p*-value	Regulation
Nrf2 (+/+) PQ10 vs. Nrf2 (+/+) Saline	1.95	1.65 × 10^−2^	Down
Nrf2 (+/+) MPTP30 vs. Nrf2 (+/+) Saline	1.79	4.42 × 10^−2^	Down
Nrf2 (- / -) MPTP30 vs. Nrf2 (- / -) Saline	7.24	4.12 × 10^−2^	Down
Nrf2 (+/+) MPTP30 vs. Nrf2 (−/−) MPTP30	3.97	4.49 × 10^−2^	Up

#### The expression intensity of miR-380-3p in the SN of Nrf2 (−/−) ICR mice after PQ or MPTP treatment

Similar to the results for the Nrf2 (+/+) mice, both MPTP treatment at 30 mg/kg and PQ treatment at 10 mg/kg decreased the expression intensity of miR-380-3p in SN of Nrf2 (−/−) ICR mice (Fig. 5b). The result is consistent with the miRNA expression profile in Nrf2 (−/−) mice.

#### Verification of miR-380-3p expression

Using real-time polymerase chain reaction (PCR), the expression of miR-380-3p in the SN in both Nrf2 (+/+) and Nrf2 (−/−) mice was confirmed. In the SN of Nrf2 (+/+) mice, the expression of miR-380-3p was upregulated by low-dose PQ treatment (*p* < 0.01), but downregulated by high-dose PQ or MPTP treatment (*p* < 0.01). A similar result was found in the SN of Nrf2 (−/−) mice; the expression level of miR-380-3p increased at a low-dose of PQ treatment (*p* < 0.01) and decreased after treatment with PQ at the high-dose or MPTP (*p* < 0.01). After MPTP treatment, the expression level of miR-380-3p was higher in the SN of Nrf2 (+/+) mice than in Nrf2 (−/−) mice (*p* < 0.01, Fig. 5c), which is consistent with the result of microarray analysis (Table 1).

#### Analysis of common target genes of miR-380-3p

**Fig. 6:**
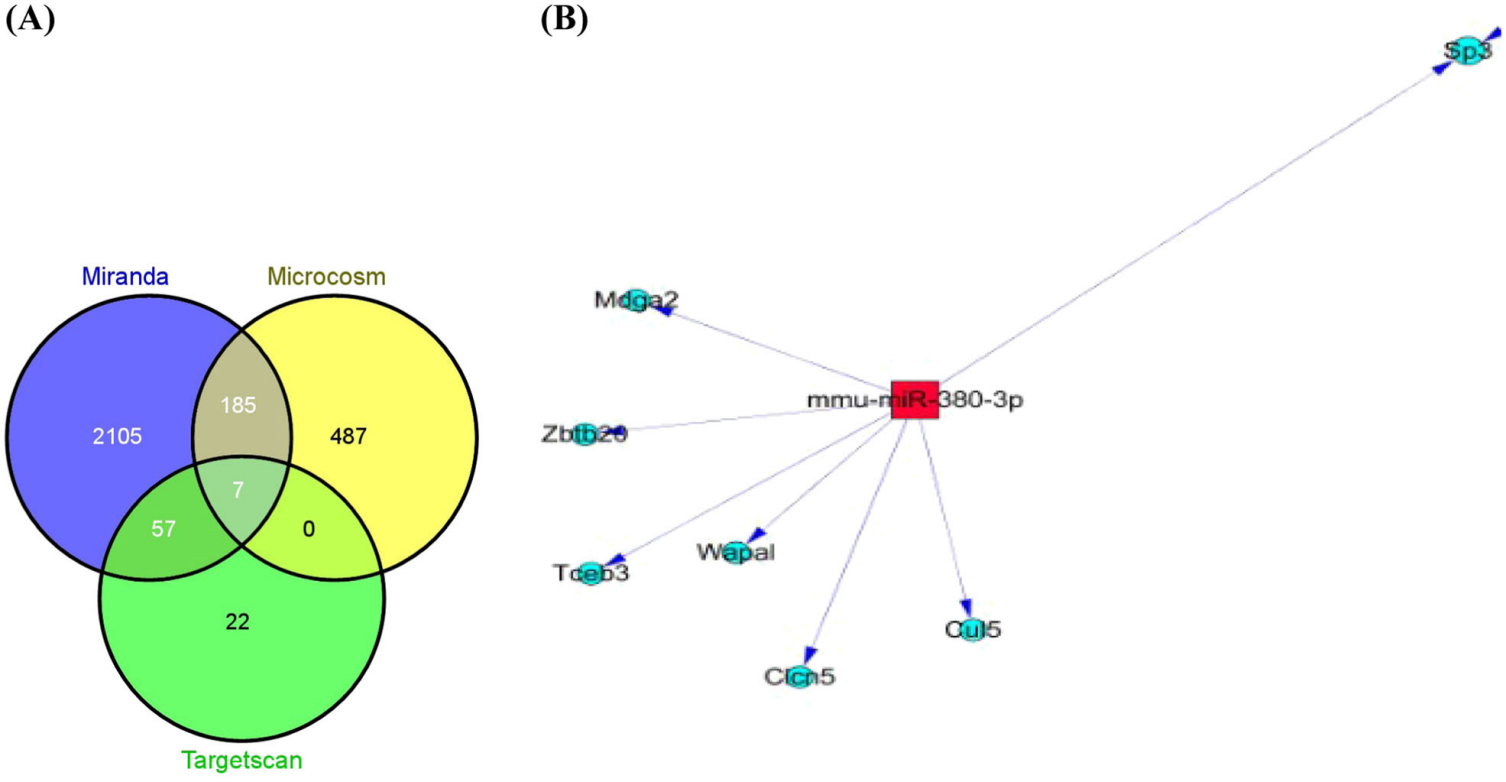
Analysis of common target genes of miR-380-3p. **a** Vennplot for the predictions of miR-380-3p target genes by Microcosm, Targetscan and Miranda. **b** The seven predicted common target genes

We predicted the target genes of miR-380-3p using Miranda, Targetscan and Microcosm software which analyze the sequences of miRNA and 3'-UTR of specific genes. All three types of software found seven common miRNA target genes (Fig. 6a, b), among which SP3 was found to be one of the target genes of miR-380-3p (Table 2).

**Table 2:** The mRNA expression level of Sp3, a suggested target gene of miR-380-3p, in the substantia nigra of Nrf2 (+/+) and Nrf2 (−/−) mice after treatment with PQ, MPTP or saline (≥2 fold, *p* < 0.05)

Contrast	Fold change	*p*-value	Regulation
Nrf2 (+/+) PQ10 vs Nrf2 (−/−) PQ10	2.05	1.44 × 10^−2^	Up
Nrf2 (−/−) MPTP30 vs Nrf2 (−/−) PQ10	3.65	1.69 × 10^−2^	Up
Nrf2 (+/+) MPTP30 vs Nrf2 (+/+) PQ10	2.26	1.35 × 10^−2^	Up
Nrf2 (+/+) MPTP30 vs Nrf2 (+/+) Saline	2.61	4.44 × 10^−2^	Up

## Discussion

MPTP-exposed mouse model is the most common model to study the mechanism of PD-related cell death because the dopaminergic neurotoxicity of MPTP can lead to DA depletion in both SN and striatum, causing behavioral change. Our present results suggest that MPTP causes regional dopaminergic neuronal damage in the mouse SN. Previous in vivo studies indicate that PQ exposure induces clinical characteristics of PD.^17–19^ In this study, we intraperitoneally administered PQ at a dose of 5 or 10 mg/kg, and each injection was followed by an interval of 2 days. After 7–8 injections of PQ at 5 mg/kg, the mice showed slight tremor, piloerection and reduced activity, while 6–7 injections at 10 mg/kg induced behavioral changes resembling those induced by MPTP. The nerve cells in the SN of mice exposed to PQ at 5 mg/kg clearly showed condensed, and the nuclei showed fragmented and a blue–black color by HE staining. However, TUNEL analysis did not find evidence of apoptosis in the cells, and the number of TH protein immunoreactive dopaminergic nerve cells was not significantly less than that in the control group . These results suggest that PQ exposure at a lower dose (5 mg/kg) did not cause significant neurotoxicity or loss of DA neurons in mice. In contrast, when the dose of PQ rose to 10 mg/kg, TUNEL analysis indicated obvious apoptosis as well as fragmented and blue–black nuclei. The number of TH positive dopaminergic neurons significantly decreased. This suggests that intraperitoneal exposure to PQ at 10 mg/kg can cause injuries to dopaminergic neurons in mouse SN. The outcome indicates a successful establishment of a PD animal model by PQ, which could be of use for further studies on mechanisms of PQ-induced neurodegeneration.

The pathogenesis of PD remains unclear, but recent studies have suggested that oxidative stress plays an important role in the process of neurodegeneration in PD.^20,21^ Many studies have shown that Nrf2 plays an important role in protecting dopaminergic neurons from damage^22,23^ induced by pesticides such as PQ,^8,24^ deltamethrin^25–27^ and manganese.^28,29^ Since loss or failed activation of Nrf2 can increase cellular sensitivity to stressors, Nrf2 may well play a protective role in PQ-induced neurodegeneration.

Nrf2 (+/+) and Nrf2 (−/−) ICR mice were used in this study to investigate the role of Nrf2 in neurodegeneration induced by PQ or MPTP. A previous study^5^ showed that MPTP at 20–60 mg/kg could cause a loss of striatal DA neurons in Nrf2 (−/−) mice. Conversely, the Nrf2 inducer D3T orally applied to wild-type mice can lead to resistance to MPTP-induced toxicity.^4^ Our results showed that 30 mg/kg of MPTP could cause cellular morphological alteration and apoptosis in the SN of either Nrf2 (+/+) or Nrf2 (−/−) mice, and the number of TH-immunoreactive DA neurons in Nrf2 (−/−) mice was significantly fewer than that in Nrf2 (+/+) mice. This finding is consistent with other researcher’s result that the number of TH-immunoreactive DA neurons in Nrf2 (−/−) mice was fewer than that in wild-type mice.^30^

It has been confirmed by a series of in vitro experiments that activation of the Nrf2 signaling pathway can protect cells from PQ-induced toxicity.^31^ Our previous in vivo studies also showed that pretreatment with *t*BHQ can enhance the expression of Nrf2 and HO-1 in SN, giving a neuroprotective effect against PQ-induced PD.^8^ Exposure to PQ at 5 mg/kg in this study turned nuclei a blue–black color and also induced nuclear condensation and fragmentation, but did not induce significant apoptosis in either Nrf2 (+/+) or Nrf2 (−/−) mice. In addition, the number of TH protein immunoreactive DA neurons was not significantly altered by exposure. These results suggest that a low-dose of PQ does not significantly impair DA neurons in mice. However, at a dose of 10 mg/kg, significant apoptosis of DA neurons was observed in both Nrf2 (+/+) and Nrf2 (−/−) mice, and there were significantly fewer TH protein immunoreactive DA neurons in Nrf2 (−/−) mice than in Nrf2 (+/+) mice. This amplification of the damage may be due to absence of the Nrf2 protein. Nrf2 protein level was reduced by a low-dose of PQ, but increased by a high-dose of PQ, suggesting that increasing the expression of the Nrf2 protein is a strategy against PQ toxicity at a high-dose. In summary, the experiments above corroborate the protective function of Nrf2 in PQ-induced neurotoxicity.

miRNAs have a significant role in the Nrf2 pathway against oxidative stress.^13,32–36^ Previous studies indicate that Nrf2 is closely associated with miRNAs in the regulatory network controlling progression of PD. In midbrain DA neurons there is a negative feedback regulation network between miR-133b and the key transcription factor Pitx3.^12^ This network constitutes the feedback loop and former feedback loop regulation of gene expression, which is an important part of the cytobiological processes in a variety of neurodegenerative diseases.^12^

To determine the role of Nrf2 in PQ- or MPTP-induced nerve cell damage, we investigated the alteration of miRNA expression profile by using Nrf2 (−/−) ICR mice with/without PQ or MPTP exposure. The main findings were summarized in Table 3, which may provide some scientific information for people to further study the mechanisms underlying PQ- or MPTP-induced neurodegeneration.

**Table 3:** Alteration of the expression profile of microRNAs in SN tissues of Nrf2 (+/+) ICR or Nrf2 (−/−) ICR mice after treatment with PQ or MPTP

Analysis	Mice group	Change miRNAs(≥1.5 fold, *p* < 0.05)	Fig.
The essential relevance between miRNA and transcription factor Nrf2.	Nrf2 (+/+) Saline—Nrf2(–/–) Saline	miR-128, miR-7a, miR-669c, miR-298, miR-543, miR-770-5p, miR-669b* and miR-544-5p	S1
Changes in miRNA expression profile after exposure to paraquat.	[Nrf2 (+/+) PQ5—Nrf2 (+/+) Saline] + [Nrf2 (+/+) PQ10—Nrf2 (+/+) Saline]	miR-140, miR-380-3p	S2a and 2b
Changes in miRNA expression profile after exposure to MPTP.	Nrf2 (+/+) MPTP30—Nrf2 (+/+) Saline	miR-140, miR-380-3p	S2c
The relationship between transcription factor Nrf2 and changes in miRNA expression profile after exposure to paraquat or MPTP.			
1.Nrf2-independent effect of 5/10 mg / kg PQ,MPTP	[Nrf2(–/–) PQ5—Nrf2(–/–) Saline] + [Nrf2(–/–) PQ10 − Nrf2(–/–) Saline] + [Nrf2 (–/–) MPTP30 − Nrf2(–/–) Saline]	miR-135b(either PQ or MPTP)	S3a, 3b and 3c
	[Nrf2(–/–) PQ5—Nrf2(–/–) Saline] + [Nrf2(–/–) PQ10 − Nrf2(–/–) Saline]	miR-142-5p and miR-451 (PQ treatment at 5/10 mg / kg)	
2.Nrf2-independent effect of MPTP	[Nrf2 (+/+) MPTP30—Nrf2(+/+) Saline] + [Nrf2 (–/–) MPTP30 − Nrf2(–/–) Saline]	miR-674*, miR-133b, miR-135b and miR-380-3p	S4a and 4b
3.Nrf2-independent effect of 5 mg / kg PQ	[Nrf2(+/+) PQ5—Nrf2(+/+) Saline] + [Nrf2(–/–) PQ5 − Nrf2(–/–) Saline]	miR-140	S5a and 5b
4.Nrf2-independent effect of 10 mg / kg PQ	[Nrf2(+/+) PQ10—Nrf2(+/+) Saline] + [Nrf2(–/–) PQ10 − Nrf2(–/–) Saline]	null	S6a and 6b
5.Nrf2-dependent effect of 5/10 mg / kg PQ	[Nrf2 (+/+) Saline—Nrf2(+/+) Saline] + [Nrf2(+/+) PQ5—Nrf2(+/+) Saline] + [Nrf2(+/+) PQ10 − Nrf2(+/+) Saline]	miR-376c, miR-154* and miR-377	S10a and 10b
6.Nrf2-dependent effect of MPTP	Nrf2 (+/+) MPTP30—Nrf2(–/–) MPTP30	miR-410, miR-379, miR-669c*, miR-467g, miR-669f-3p, miR-1902, miR-615-3p, miR-99b*, miR-138-1, miR-378/miR-378b, miR-351, miR-let-7d*, miR-34b-3p, miR-543, miR-376c* and miR-380-3p.	S7
7.Nrf2-dependent effect of 5 mg / kg PQ	Nrf2(+/+) PQ5—Nrf2(–/–) PQ5	miR-135a, miR-376c, miR-31, miR-let-7i*, miR-669b*, miR-344, miR-15b, miR-700*, miR-3099, miR-377, miR-338-5p, miR-382, miR-219-3p and miR-310a	S8
8.Nrf2-dependent effect of 10 mg / kg PQ	Nrf2(+/+) PQ10—Nrf2(–/–) PQ10	miR-495*, miR-154*, miR-let-7b, miR-1983, miR-103 and miR-26a	S9

The miR-380-3p / Sp3 mRNA pathway is worth to mention here. We have found previously that expression of miR-380-3p was altered in lithium–pilocarpine-induced epilepticus models of Sprague–Dawley rat and in models of temporal lobe epilepsy.^37^ Current study investigating the link between Nrf2 and miR-380-3p found that expression of miR-380-3p was not altered until PQ dose rose to 10 mg/kg (fold difference = 1.95) or MPTP dose to 30 mg/kg (fold difference = 1.79). However, independent of Nrf2, 30 mg/kg of MPTP could cause changes in expression of miR-380-3p (difference ratio = 7.24), suggesting that miR-380-3p expression may not only depend on Nrf2. In fact, expression of miR-380-3p was found to be altered by the Nrf2-MPTP interaction effect (fold difference = 3.97). The expression of miR-380-3p was also verified by LNA–ISH and quantitative reverse transcription PCR (QRT–PCR), which is consistent with the expression profiling. These results indicate that miR-380-3p may be involved in the neurotoxicity induced by PQ or MPTP, and that MPTP may cause changes in miRNA expression profile via interaction with Nrf2.

Furthermore, we predicted the mRNAs targeted by miR-380-3p using three target gene prediction programs and analyzed the possible biological effects. By seeking common intersection of results from the three programs, we found seven common target genes (Sp3, Cu5, Clcn5, Wapal, Tceb3, Zbtb20 and Mdga2). We compared the predicted results with the results of our mRNA chips and found that Sp3 mRNA level was altered by miR-380-3p. Sp3 is one of the basic transcription factors involved in virtually all cellular functions including cell proliferation, apoptosis and differentiation.^38^

The results showed that there may be interaction between MPTP and Nrf2, which could lead to changes in miRNA expression profile in the SN. It can be speculated that MPTP may interact with Nrf2 in the SN, thereby regulating the expression of miR-380-3p. miR-380-3p could then alter the mRNA level of Sp3, thus affecting the biological function of the cells, inducing neurotoxicity. Therefore, the miR-380-3p/Sp3 mRNA pathway may be part of the mechanism of MPTP-induced neurotoxicity, although it requires further experimental verification.

Where Nrf2 interacted with both low-dose and high-dose PQ, the miRNA expression profile altered differentially at different doses of PQ. Since low-dose PQ did not significantly impair DA neurons, while high-dose PQ caused a significant loss of TH positive dopaminergic neurons, suggesting that changes in the expression profile vary depending on the dose of PQ and damage extent by its toxic effect. In Nrf2 (+/+) mice, Nrf2 protein level was reduced by low-dose PQ but increased by high-dose PQ, suggesting that it might be a natural strategy against the toxic effects of high doses of PQ to increase the amount of Nrf2 protein. Therefore, alteration of miRNAs by high-dose PQ where PQ interacted with Nrf2 may well play a crucial role in the resistance to the toxic effects of high doses of PQ. In summary, the major findings of the study were as follows: (1) In vivo experimental results corroborate the protective role of Nrf2 in PQ-induced neurodegeneration. (2) In vivo exposure to PQ or MPTP changed miRNA expression profile, which may be part of the mechanism of neurodegeneration of PQ or MPTP. (3) In the SN, the interaction of Nrf2 and MPTP could cause alteration in miRNA expression profile. This could in turn regulate the miR-380-3p/Sp3-mRNA pathway, which is likely part of the mechanism of MPTP-induced neurodegeneration. (4) Changes in miRNA expression profile in the brain caused by PQ may involve its interaction with Nrf2, and the alteration varies according to the dose of PQ. The results may provide evidence on underlying mechanisms of PQ-induced or MPTP-induced neurodegeneration. Understanding the role of transcription factor Nrf2 on neurodegeneration and alteration in the expression profile of microRNAs induced by PQ or MPTP in vivo may provide insights into more effective preventive and therapeutic approaches for the PD. Thus, on the basis of our current study, an intriguing orientation for further research has been proposed, and the exact and detailed mechanism studies in the future are expected.

## Materials and methods

### Reagents

PQ dichloride hydrate (99.2% w/w) and MPTP (99.99% w/w) were purchased from Sigma-Aldrich Co. (St. Louis, MO). The terminal deoxynucleotidyl transferase-mediated dUTP biotin nick end-labeling (TUNEL) kit and Maxvision^TM^ (HRP-polymer anti-rabbit IHC kit) were purchased from Fuzhou Maixin Biotech. Co. Ltd. (China). Anti-Nrf2 (C-20, *sc-722*) and anti-TH (*sc-374047*) antibodies were purchased from Santa Cruz Biotechnology Inc. (Santa Cruz, CA). A Bulge-Loop^TM^ miRNA QRT–PCR Primer kit was purchased from RiboBio Co. Ltd. (Guangzhou, China). A Primer Script RT Reagent Kit (Perfect Real-Time) kit and a SYBR Premix Ex Taq II kit were purchased from TaKaRa Biotechnology Co. Ltd. (Japan). A MicroRNA ISH Buffer, Controls Kit, and a LNA™ microRNA-380-3p probe were purchased from Exiqon Life Sciences, Life Sciences and Diagnostics Co. (Denmark). QRT–PCR primers for microRNA-380-3p were purchased from Guangzhou RiboBio Co., Ltd (China). Goat anti-Rabbit-DIG-AP IgG and NBT/BCIP staining solutions were purchased from Roche Co. (USA). Sheep serum was purchased from Millipore Corporation (Temecula, CA).

### Animals, PQ administration, MPTP administration and dissection of brain tissues

Although C57/BL6 mice are the most MPTP-sensitive strain, the only strain of Nrf2 knockout mice available is ICR, obliging us to use this strain in the current experiment. The reason for selecting male mice is that MPTP fatality rate is higher in females. All mice weighed 18–22g and were 6–8 weeks of age, consistent with previous studies.^17^ Forty-eight Nrf2 (+/+) ICR and Nrf2 (−/−) ICR male mice were obtained from Johns Hopkins University (USA). Mice were housed in cages at an ambient temperature of 20–25 °C under a 12-h light/dark cycle, and allowed free access to food and water. The experimental protocols were in accordance with the National Institutes of Health Guide for the Care and Use of Laboratory Animals, and were carried out with the approval of the Animal Use Committee, Fujian Medical University. Efforts were made to minimize animal suffering.

The 48 mice were equally divided into eight groups, Nrf2 (+/+) saline, Nrf2 (+/+) PQ5, Nrf2 (+/+) PQ10, Nrf2 (+/+) MPTP30, Nrf2 (−/−) saline, Nrf2 (−/−) PQ5, Nrf2 (−/−) PQ10, and Nrf2 (−/−) MPTP30. The Nrf2 (+/+) saline and Nrf2 (−/−) saline groups were intraperitoneally injected with saline. The Nrf2 (+/+) PQ5 and Nrf2 (−/−) PQ5 groups were injected with 5 mg/kg PQ dichloride hydrate (dissolved in saline) at 2-day interval for a total of ten doses. The Nrf2 (+/+) PQ10 and Nrf2 (−/−) PQ10 groups were injected with 10 mg/kg PQ dichloride hydrate (dissolved in saline) at 2-day intervals for a total of ten doses as PD animal models.^8,39^ The Nrf2 (+/+) MPTP30 and Nrf2 (−/−) MPTP30 groups were administrated with 30 mg/kg MPTP (dissolved in saline) by hypodermic injection every day for 5 days as classic PD animal models.

For the neurobehavioral, histological and TUNEL analyses, the mice were executed under anesthesia 7 days after the last administration. The mice were anesthetized with 0.5% sodium pentobarbital and perfused via a left ventricular puncture of the heart with cold 0.9% saline (4 °C), followed by 4% paraformaldehyde. The brains were removed and stored overnight in 10% formalin solution. The coronal section (6 µM thick) passing through the SN was cut and then embedded in paraffin. For the immunoblot analysis, the mice were executed under anesthesia 4 h after the last administration. The brain tissue was immediately removed, and SN tissue was obtained from the coronal slices.^40^ The dissected brain tissue was immediately frozen and stored at −80 °C until further processing.

### TUNEL assay

The formalin-fixed, paraffin-embedded tissue samples were sectioned into 6 µM-thick slices using a microtome. The tissue sections were deparaffinized, rehydrated, and stained using the TUNEL kit according to the manufacturer’s instructions. The following items were considered to be an indication of apoptosis: (a) marked condensation of chromatin and cytoplasm (apoptotic cells); (b) cytoplasmic fragments with or without condensed chromatin (apoptotic bodies) and (c) intracellular and extracellular chromatin fragments (micronuclei). Using these criteria significantly reduced the effect of nonspecific staining on determining TUNEL-positive cells and apoptotic cells. The TUNEL-positive cells and apoptotic cells were counted in each section through the SN. Three sections containing the same position of SN were chosen. The extent of brain damage was evaluated by the apoptotic index, which was the average percentage of TUNEL-positive cells in each section counted in 10 microscopic fields (×400 magnification).

### Immunohistochemical staining

The entire brain was sliced using a sliding microtome into 6 µM consecutive sections. Every third section was immunohistochemically processed for TH and Nrf2. For TH staining, anti-mouse TH antibody (diluted at 1:300) and a MaxVision TM HRP-polymer anti-rabbit IHC kit were used. Three tissue slices containing the same position of SNc were obtained from the head end, middle part and the tail end of SN, respectively, and the TH staining positive neurons on both sides of an area above the midpoint of pedunculus cerebri were counted at high magnification of ×100. And the numbers obtained from three slices were averaged. The scorer was blinded to the treatment groups. In addition, the tissue sections were used for immunohistochemical staining, which was performed using anti-Nrf2 (diluted at 1:400) antibodies. Sections incubated in the absence of a primary antibody were used as negative controls. Six views were selected randomly for each section and observed under a light microscope (100 and ×400 magnification).

### Microarray analysis of miRNA expression

RNA extraction: Total RNA was isolated using Trizol (Invitrogen) and a miRNeasy mini kit (QIAGEN) according to the manufacturer’s instructions, which efficiently recovered all RNA species including miRNAs. RNA quality and quantity was measured using a nanodrop spectrophotometer (ND-1000, Nanodrop Technologies) and RNA integrity was determined by gel electrophoresis.

#### RNA labeling

After RNA isolation from the samples, the miRCURY™ Hy3™/Hy5™ Power labeling kit (Exiqon, Vedbaek, Denmark) was used according to the manufacturer’s guidelines for miRNA labeling. A 1 µg sample was 3′-end-labeled with a Hy3™ fluorescent label, using T4 RNA ligase, by the following procedure: RNA in 2.0 μL of water was combined with 1.0 μL of CIP buffer and CIP (Exiqon). The mixture was incubated for 30 min at 37 °C, and was terminated by incubation for 5 min at 95 °C. Then 3.0 μL of labeling buffer, 1.5 μL of fluorescent label (Hy3™), 2.0 μL of DMSO and 2.0 μL of labeling enzyme were added to the mixture. The labeling reaction was incubated for 1h at 16 °C, and terminated by incubation for 15 min at 65 °C.

#### Array hybridization

Following termination of the labeling procedure, the Hy3™-labeled samples were hybridized to the miRCURY™ LNA Array (v.16.0, Exiqon) according to the array manual 25 μL of the mixture from Hy3™-labeled samples with 25 μL hybridization buffer were first denatured for min at 95 °C, incubated on ice for 2 min, and then hybridized by the microarray for 16–20 h at 56 °C in a 12-Bay Hybridization System (Hybridization System–Nimblegen Systems, Inc, Madison, WI, USA). This provided an active mixing action and constant incubation temperature to improve hybridization uniformity and enhance the signal. Following the hybridization, the slides were washed several times using a wash buffer kit (Exiqon), and finally dried by centrifugation for 5 min at 400 rpm. The slides were subsequently scanned using an Axon GenePix 4000B microarray scanner (Axon Instruments, Foster City, CA).

#### Heat map and hierarchical clustering

The heat map diagram shows the result of the two-wayhierarchical clustering of miRNAs and samples. Each row represents a miRNA and each column represents a sample. The miRNA clustering tree is shown on the left, and the sample clustering tree appears at the top. The color scale shown at the top illustrates the relative expression level of a miRNA in the certain slide: red color represents a high relative expression level, green color represents a low relative expression levels. According to Heat Map and Hierarchical Clustering, we chose miRNA whose folder change ≥1.5, *p* < 0.05 associatting with their biological significance.

### miRNA quantitative reverse-transcription PCR

Based on the results of the miRNA array, selected miRNAs were validated by SYBR Green I miRNA assays using a Lighter 480 Real-Time PCR System (Roche, USA). The miRNAs were selected based on the greatest fold change and a significant difference between the probe and the background signal. Briefly, 1 μg of small RNA was reverse transcribed using a miRNA cDNA Synthesis Kit and primers. The cycle parameters for the reverse transcription (RT) reaction were 42 °C for 15min and 85 °C for 5 s followed by maintenance at −20 °C. Following the RT reactions, 2 μL of complementary DNA was used for PCR. The PCR was conducted at 95 °C for 30 s, followed by 40 cycles of 95 °C for 5 s and 60 °C for 34 s in an ABI 7500 real-time PCR System. The results of real-time PCR were analyzed and expressed as a relative miRNA level, using the U6 small nuclear RNA for normalization; the threshold cycle (Ct) range for the control U6 small RNA ranged from 11 to 15 cycles. The delta Ct (ΔCt) values in each sample represented the relative expression amount of miRNA: ΔCt = Ct (miRNA) − Ct (U6). The fold expression changes between groups were determined using the comparative Ct method (2−^ΔΔCT^). Cells treated with saline were used as calibrator samples. All experiments were performed in triplicate.

### Locked nucleic acid in situ hybridization (LNA–ISH)

LNA–ISH was performed on using LNA™ probes for miR-380-3p (Exiqon, Woburn, Mass, USA), in order to validate the results of miR-380-3p in miRNA expression profile. Briefly, after de-paraffinization the slides were incubated in proteinase K solution and then in 0.2% glycine. Next, the slides were fixed in 4% paraformaldehyde and then washed in phosphate-buffered saline (PBS). After rinsing in PBS, the slides were pre-hybridized with hybridization buffer for 2 h at room temperature, and then incubated with hybridization buffer containing the digoxigenin-labeled LNA™ probe in an oven at 48 °C overnight. A parallel set of SNs was hybridized with a scrambled miRNA probe (negative control; Exiqon). After stringent washes with 50% formamide and 2× SSC at 53 °C, the slides were blocked with blocking buffer for 1 h and incubated with anti-digoxigenin Fab fragment (1:2000) overnight in a humid chamber at 4 °C. The colorimetric detection reaction was performed using NBT/BNIP staining solution (Roche, USA) in the dark for 48 h. After stringent washes, the slides were stained with Mayer hematoxylin, and then mounted with cover-slips using neutral resins. Six views were selected randomly for each section and observed under an Olysia-BioReport imaging system (Olympus Corporation, Japan) (×400 magnification).

### Bioinformatic analysis of miRNA-380-3p and comparison

The expression microarray of miRNAs provided a large variety of the gene list involved in oxidative stress. In our study, we selected miRNA-380-3p for computational prediction using Microcosm (www.ebi.ac.uk/enright-srv/microcosm/htdocs/targets/v5/), Targetscan (www.targetscan.org/vert_60/), and Miranda (www.microrna.org/microrna/home.do). We then compared the predicted target genes with microarray analysis of mRNA in order to find the common target gene.

### Statistical analysis

Each data bar represents the mean values ± SD of at least three independent experiments in all cases. Results were analyzed using SPSS for Windows (version 19.0). Differences between groups were analyzed by analysis of variance. If the *F* values were significant, least significant difference post hoc tests were used to compare multiple groups. A *p* value of <0.05 was considered statistically significant in all cases.

### Data availability statement

All relevant data are within the paper and its Supporting Information files.

## Electronic supplementary material

Supplemental figures
