# Supplementary material for: Paraquat and MPTP induce neurodegeneration and alteration in the expression profile of microRNAs: the role of transcription factor Nrf2
Source: NPJ Parkinsons Dis. 2017 Oct 20;3:31. doi: 10.1038/s41531-017-0033-1 (PMC5651826; doi:10.1038/s41531-017-0033-1)
Supplement: Supplementary file 1 — Supplemental figures [file 41531_2017_33_MOESM1_ESM.doc]

**Paraquat and MPTP induce neurodegeneration and alteration in the expression profile of microRNAs: the role of transcription factor Nrf2**

**Qingqing Wang^1,2^*,* Nan Ren^1^*,* Zhipeng Cai^1^****, Qingxia Lin^1^, Zhangjing Wang^1^, Qunwei Zhang^3^*,***  **Siying Wu^4,*^ , Huangyuan Li^1,*^**

^1^Department of Preventive Medicine, Fujian Provincial Key Laboratory of Environment Factors and Cancer, School of Public Health, Fujian Medical University, Fuzhou 350122, China, ^2^Zhangzhou Entry-Exit Inspection and Quarantine Bureau, Shicangduan, Shuixian Street, Zhangzhou 363000, China, ^3^Department of Environmental and Occupational Health Sciences, University of Louisville, 485 E. Gray Street, Louisville, KY 40202, USA. ^4^Department of Epidemiology and Health Statistics, Fujian Provincial Key Laboratory of Environment Factors and Cancer, School of Public Health, Fujian Medical University, Fuzhou 350122, China. Correspondence and requests for materials should be addressed to S.Wu(email:fmuwsy[@163.com](mailto:fmulhy@163.com)) or H.Li(email:[fmulhy@163.com](mailto:fmulhy@163.com))

**Short title: Neurodegeneration and altered expression of miRNAs induced by**  **neurotoxicants and Nrf2**

**Supplemental figures**

Ap Nrf2 (+/+) Saline, Am Nrf2 (-/-) Saline, Bp Nrf2 (+/+) PQ5, Bm Nrf2 (-/-) PQ5, Cp Nrf2 (+/+) PQ10, Cm Nrf2 (-/-) PQ10, Dp Nrf2 (+/+) MPTP30, Dm Nrf2 (-/-) MPTP30


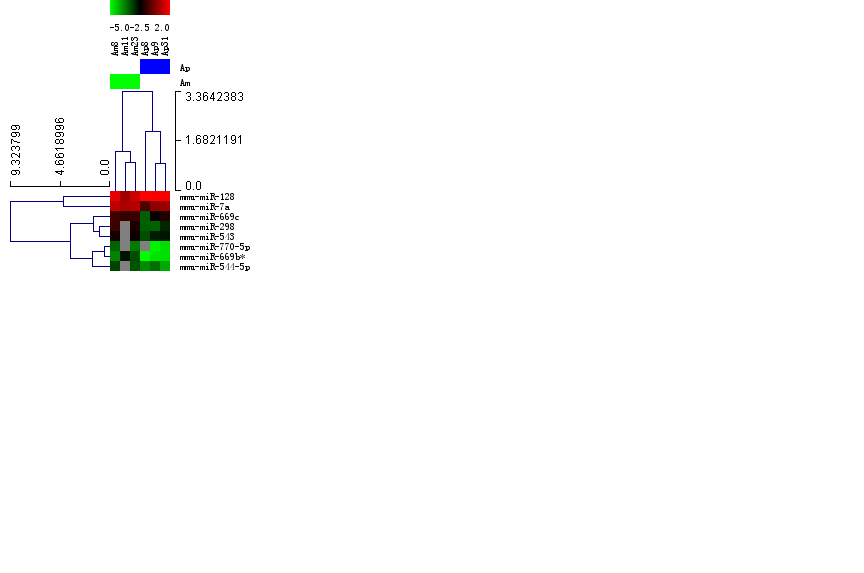


**Figure-S1. The heat map of differentially expressed miRNAs in the substantia nigra of Nrf2 (+/+) or Nrf2 (-/-) mice treated with saline.**

**
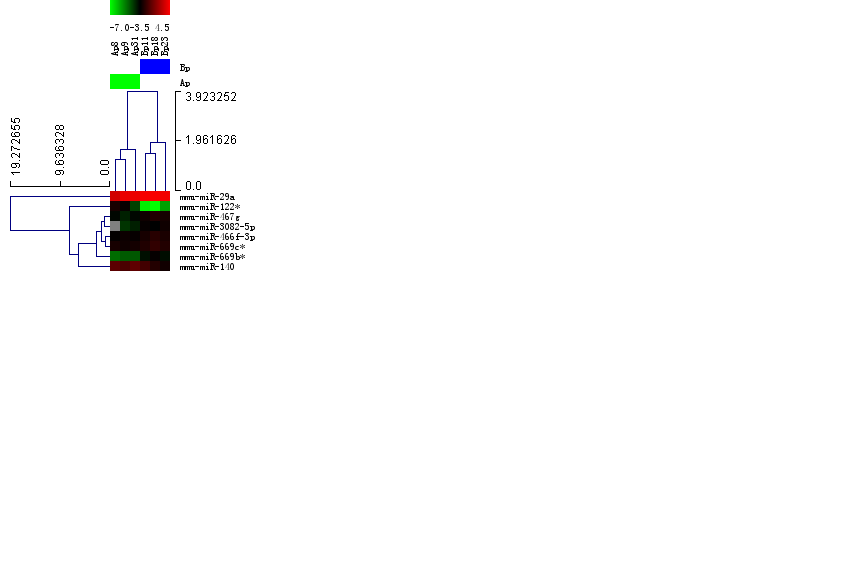
**

**Figure-S2a. miRNAs which were differentially expressed in the substantia nigra of Nrf2 (+/+) ICR mice treated with 5 mg / kg PQ or saline.**


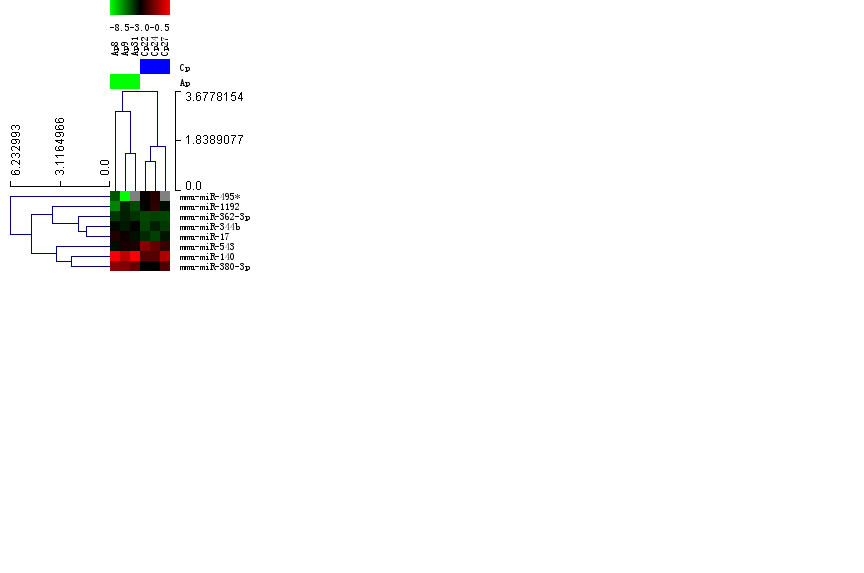


**Figure-S2b. miRNAs which were differentially expressed in the substantia nigra of Nrf2 (+/+) ICR mice treated with at 10 mg / kg PQ or saline.**

**
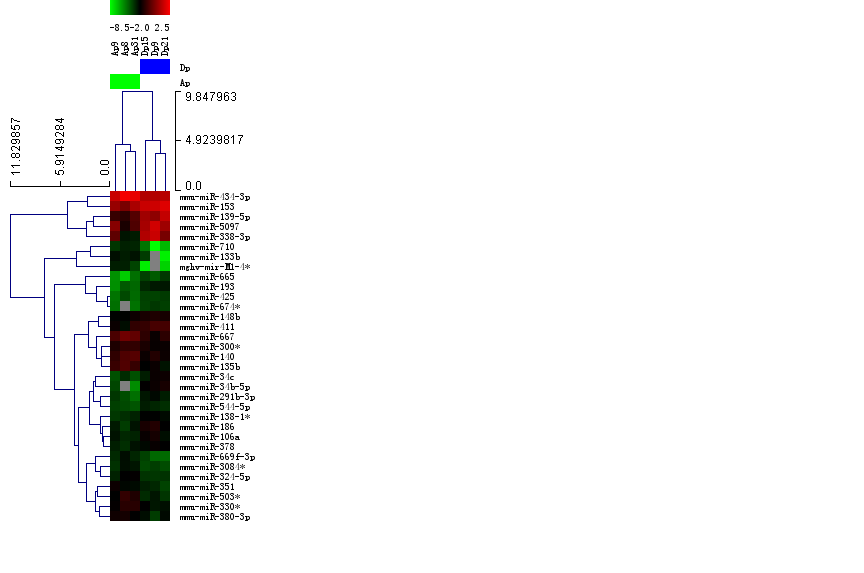
**

**Figure-S2c. miRNAs which were differentially expressed in the substantia nigra of Nrf2 (+/+) ICR mice treated with MPTP at 30 mg / kg or saline.**

**
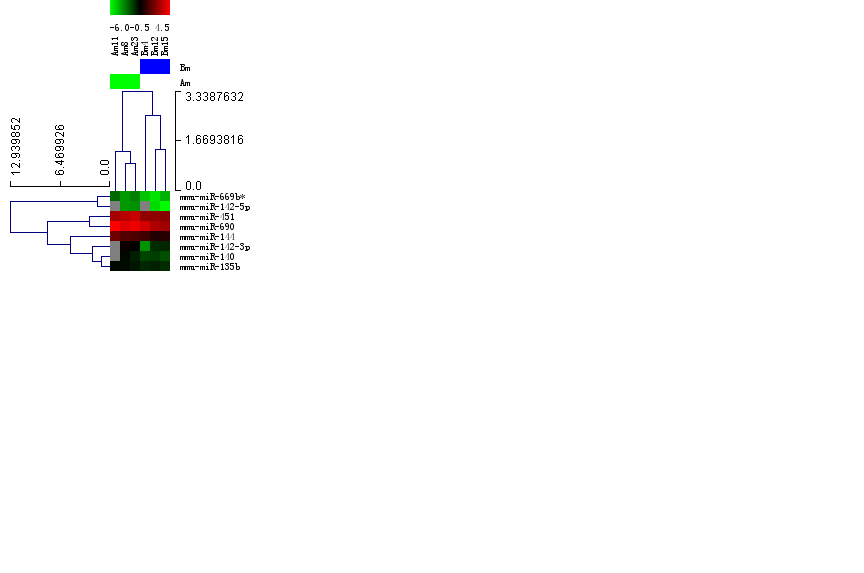
**

**Figure-S3a. The expression of miRNAs was altered after exposure to PQ at 5 mg / kg in the substantia nigra of Nrf2 (-/-) ICR mice compared to those exposed to saline.**


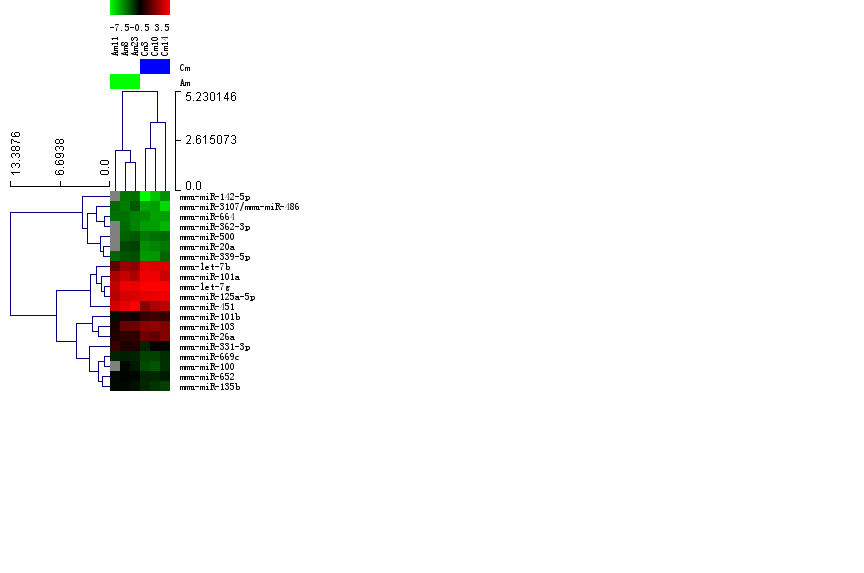


**Figure-S3b. The expression of miRNAs was altered after exposure to PQ at 10 mg / kg in the substantia nigra of Nrf2 (-/-) ICR mice compared to those exposed to saline.**


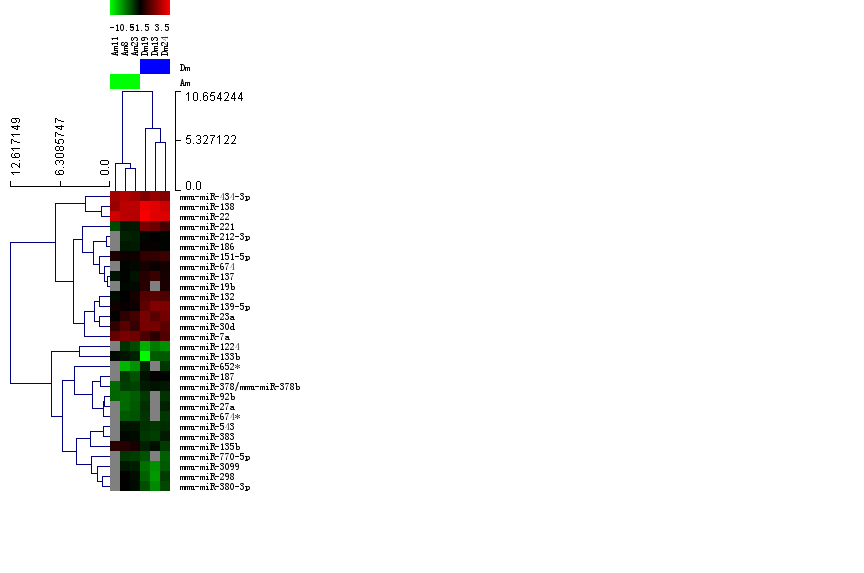


**Figure-S3c. The expression of miRNAs was altered after exposure to MPTP at 30 mg / kg in the substantia nigra of Nrf2 (-/-) ICR mice compared to those exposed to saline.**


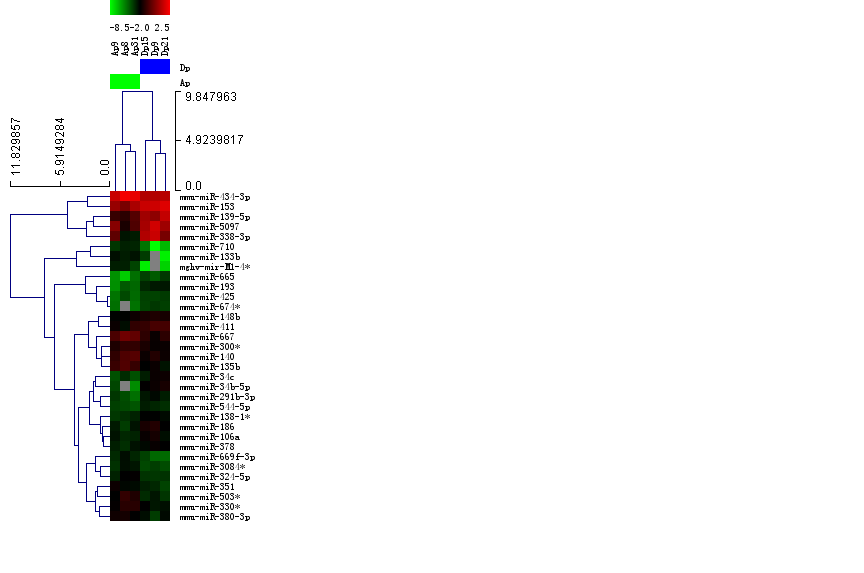


**Figure-S4a. The expression of miRNAs was altered after exposure to MPTP at 30 mg / kg in the substantia nigra of Nrf2 (+/+) ICR mice compared to those exposed to saline.**


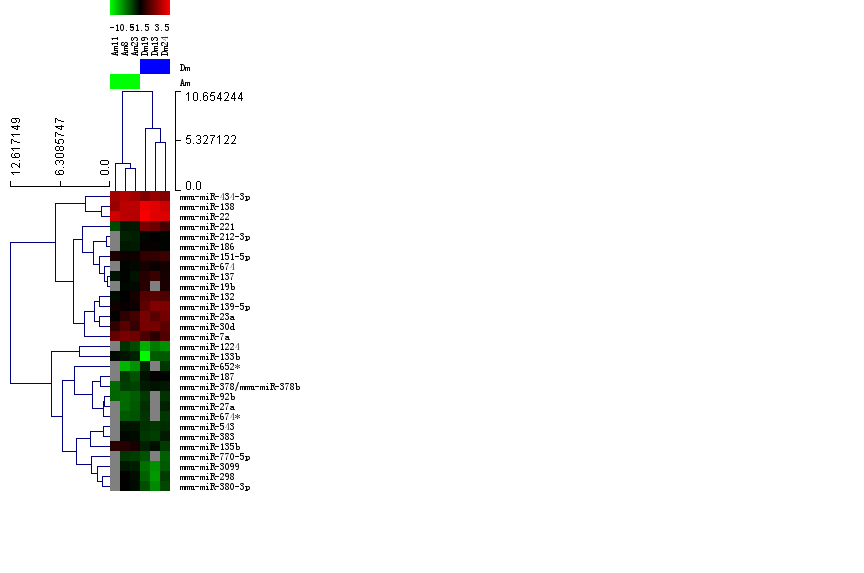


**Figure-S4b. The expression of miRNAs was altered after exposure to MPTP at 30 mg / kg in the substantia nigra of Nrf2 (-/-) ICR mice compared to those exposed to saline****.**


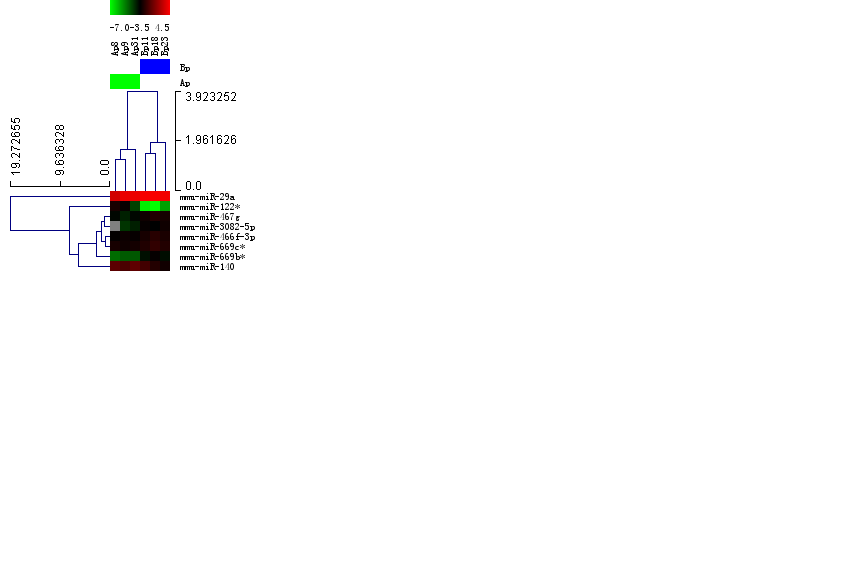


**Figure-S5a.** T**he expression of miRNAs was altered after exposure to PQ at 5 mg / kg in the substantia nigra of Nrf2 (+/+) ICR mice compared to those exposed to saline.**


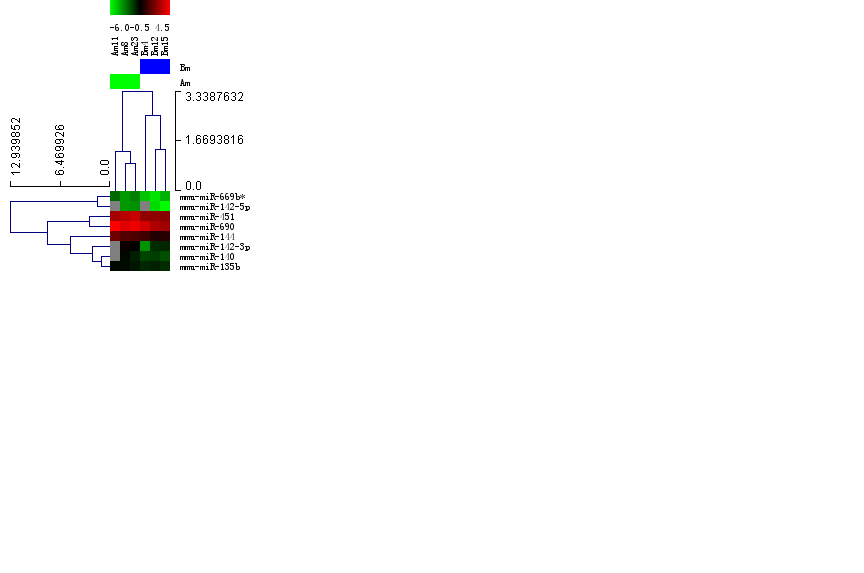


**Figure-S5b. The expression of miRNAs was altered after exposure to PQ at 5 mg / kg in the substantia nigra of Nrf2 (-/-) ICR mice compared to those exposed to saline.**


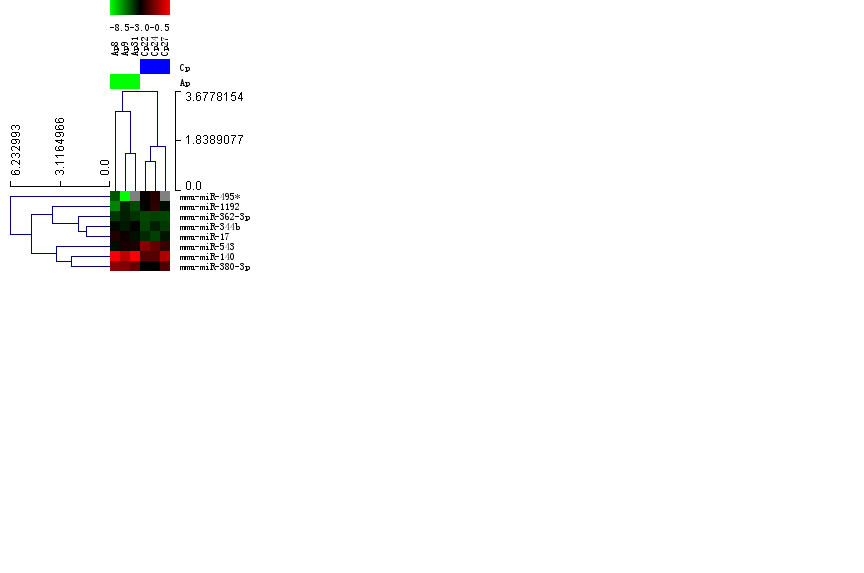


**Figure-S6a. The expression of miRNAs was altered after exposure to PQ at 10 mg / kg in the substantia nigra of Nrf2 (+/+) ICR mice compared to those exposed to saline.**


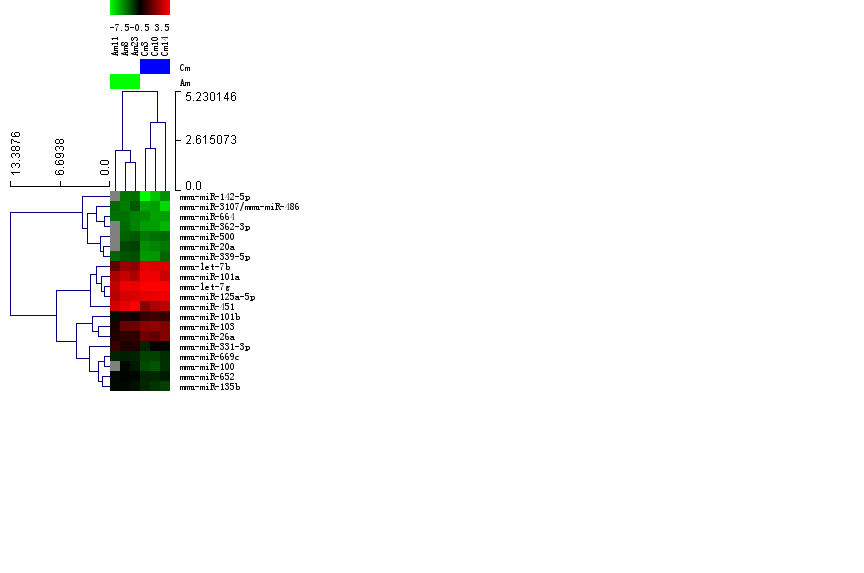


**Figure-S6b. The expression of miRNAs was altered after exposure to PQ at 10 mg / kg in the substantia nigra of Nrf2 (-/-) ICR mice compared to those exposed to saline.**


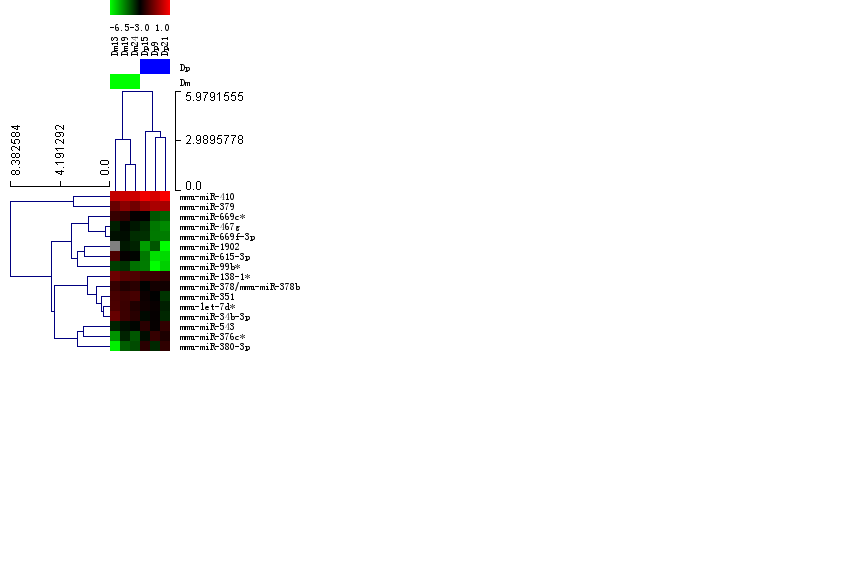


**Figure-S7. A difference was found in miRNA expression between Nrf2 (-/-) and Nrf2 (+/+) mice when both types were exposed to exposed to MPTP at 30 mg / kg.**


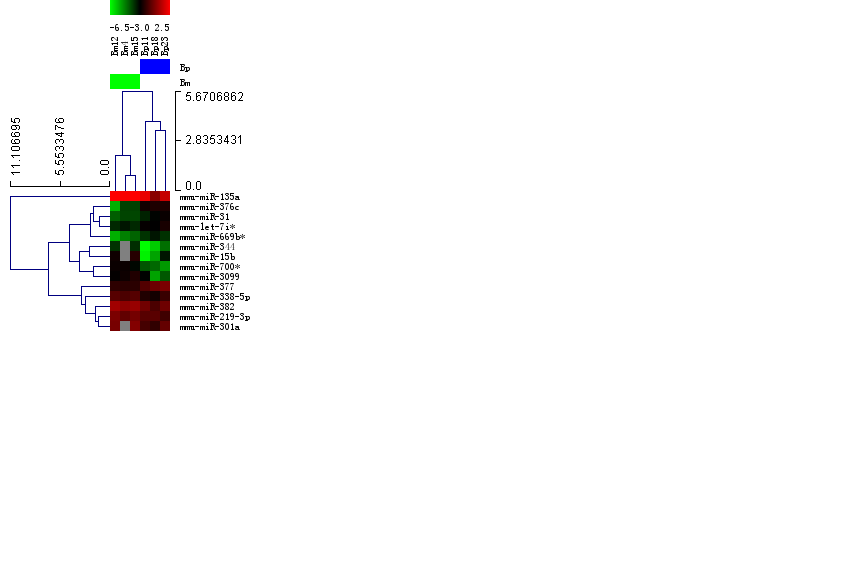


**Figure-S8. A difference was found in miRNA expression between Nrf2 (-/-) and Nrf2 (+/+) mice when both types were exposed to exposed to PQ at 5 mg / kg.**


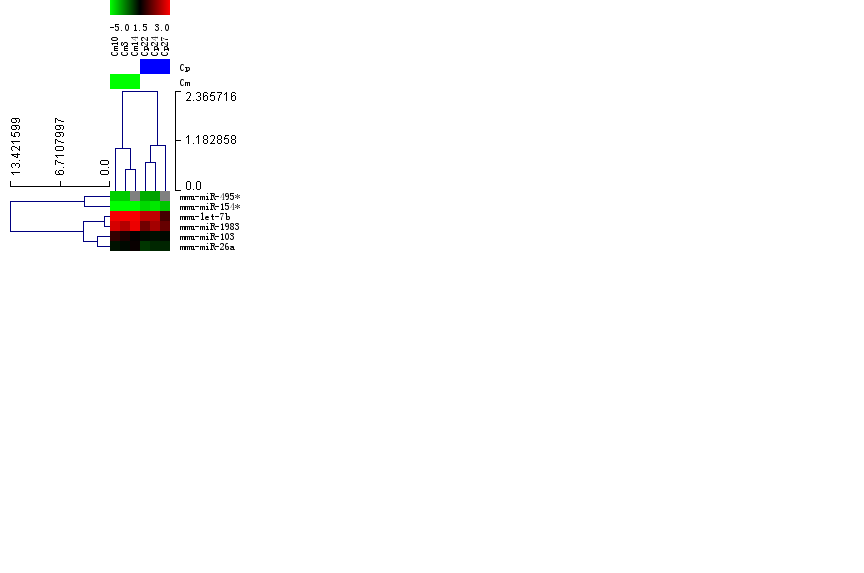


**Figure-S9. A difference was found in miRNA expression between Nrf2 (-/-) and Nrf2 (+/+) mice when both types were exposed to PQ at 10 mg / kg.**


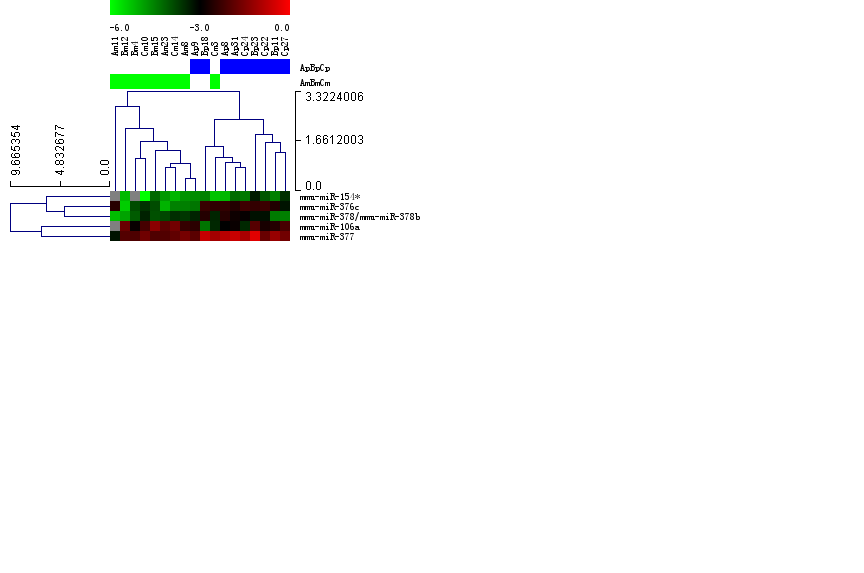


**Figure-S10a. A difference was found in miRNA expression profile between Nrf2 (-/-) and Nrf2 (+/+) mice after treatment with PQ or saline.**


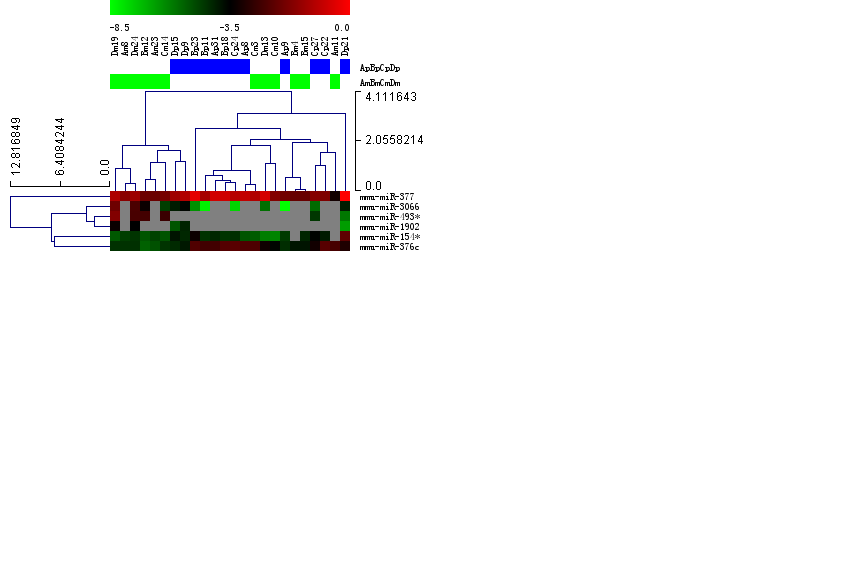


**Figure-S10b. A difference was found in miRNA expression profile between Nrf2 (-/-) and Nrf2 (+/+) mice after treatment with MPTP, PQ or saline.**
